# From Our Foreign Correspondents

**Published:** 1987-02

**Authors:** 


### From Our Foreign Correspondent

### Educational visit to Paris by 'Onze Medecins Britanniques'

Attracted by such divers siren calls as advertisements in the London Physicians' College Commentary, the BMJ, and glowing reports from past élèves, a miscellaneous group of British doctors found themselves together for a week's residential French course in Paris. We numbered two haematologists, two surgeons, two physicians, four GP's and one spouse. We came from all four corners of our islands (if you could call Cheshire Wales!), and there were three old-stagers and eight fresh persons.

We met up at the most impressive château-type 'Centre International d'Etudes Pedagogiques' at Sèvres, a Metro terminus on the south west edge of Paris. The building was of considerable historical interest, having been commissioned by Madame de Pompadour as the original porcelain factory, subsequently serving as a teacher training college, then suffering wartime occupation before being adapted to its present rôle. Its interior was as spartan as the exterior was grand, and trudging up four flights of stairs to our rooms (lift to be installed next year), memories of school and university came flooding back, to set us all in a most apt frame of mind for the coming week. The rooms in the Mansard garrets were clean and functional (but a long long way from ground level). The two public telephones in the front hall lay under permanent siege by the polyglot inhabitants of the Centre.

Dinner on Sunday night was a self-service cold table affair, enlivened and extended by our Irish representative finding the central wine store unlocked. The standard house red and white wines were delightfully light, tasty, and truly innocuous. Indeed, one could cheerfully consume several glasses twice a day without developing any need for recurrent siestas.

At breakfast next day (8.30 am) we severally made the interesting discovery that the British docs were a tiny minority among some 120 vociferous French-speaking fellow students. Resolutely refusing to sit together as a group (and speak English!), our halting but improving French soon led us to discover that we were onze medecins Britanniques among some cent-vingt professeuses étranges de la langue Francais. They came from Sweden, Denmark, Finland, Belgium and several other countries (even a few from England) and were each doing a three-week course (or 'stage') at the Centre, to brush up their French and teaching methods.

They certainly made the most splendid company. To a person, they were incredulous that any Englishman was actually bothering to learn someone else's language. (Dr Johnson's statement about the dog walking on its hind legs comes to mind.) But, this accepted, they were bursting with enthusiasm to guide our faltering linguistic footsteps. Indeed, one could not but contrast the sort of non-welcome a first-aider might expect at a medical conference, with the tolerant attention our fellow stagiaires (that's the French word) gave to our puny malconstructed French sentences: the gulfs in knowledge and performance being of a similar order. Perhaps we were benefiting from a combination of the protective instinct and the developed need to teach, common to pedagogues the world over. However, French it was, and French it had to be, if one was not to starve for lack of victuals.

So we finally assembled for our first class, in the echoing formal library, a very grand 18th century room complete with a first edition of Diderot's encyclopaedia, and having the acoustics of an empty swimming pool (provoking memories of the language test in the ECFMG). There we met our teachers, who maintained stoutly, until the last morning, that they hardly understood a word of English, whereas, in fact, they spoke it as fluently and perhaps more idiomatically, than we did. We discovered that the mornings were to be devoted to 'perfectionner la langue' (what a joyously ludicrous overstatement of our aspirations!), and the afternoons would be free, expect for one hospital visit. Special trips could be made by arrangement, lunch was at 1.30 pm, dinner at 6.45 pm and here was a map of the Metro. After our opening session and a guided tour of the building (where Madam Curie was once a teacher), we gathered for our mid-morning Muscat wine and biscuits. The more abstemious could take simple apple juice. And so back to our classes, in a more cosy seminar room, until lunch at 1.30 pm. On our first day this was preceded by a little party of welcome, at which Madame la Directrice announced that the excellent champagne she was serving was another product of the French educational establishment—in this case the agricultural college near Rheims.

As the week wore on, we settled into a pleasurable routine of video-presentation, discussions and French conversation (in groups of five). It transpired that our group encompassed a considerable range of achievement, or non-achievement, in la langue Francaise, but we all improved—the good to better, the poor to moderate and the bad to poor! The teaching was about one third medical and two thirds general, although the hospital visits were, naturally, very medical. As every teacher/examiner will know, one of the main intentions is to keep the discussion on a level and within bounds that are familiar to the mentor; thus we were thrilled to hit gold when the conversation turned to haute cuisine.

Now it is a strange thing, but very true for all that, that the French hold their own grande cuisine in such awe, and place it on such a pedestal, that relatively few of them have much to do with it on a personal basis. Not so for their group of Francophile Brits who, to a person, found that here at least they were on solid ground. They had the knowledge, they had the vocabulary, and they had the experience, so it rapidly transpired that the taught were away out in front of the teachers, calling the tune and knowing the answers. It couldn't last, it didn't last, but it was a happy interlude. Dinner in the evening (6.45 pm) was convivial. The food was good, the wine flowed, our Saxon tongues were loosened, and our French improved.

The days sped by. Our teachers went to great trouble to arrange appropriate visits for us to colleagues in our specialties. One and all were well received, the surgeons, haematologists and accident doctors doing particularly well. Our group visit one afternoon to Hôpital St Louis near the Bastille was a most interesting experience for us all, with a chance to see an excellent example of the French flair for juxtaposition of the old and the ultra-modern. A really well-conceived, well built, and attractive modern hospital, set in the grounds of a beautiful, and sympathetically restored sixteenth century one. None of us will forget the eerie sight of food trolleys wandering unattended and 'Dalek-like' towards their destination.

Betweenwhiles we visited Paris according to our several interests. The Renoir Exhibition at the Grand Palais was marvellous, and devotees explored the Jeu des Paume and the Pompidou Centre. On the last Saturday night we went to a small restaurant near the Latin quarter, recommended by one of our teachers, and dined splendidly in central Paris for £13 a head (tout compris). Later we drank Eau de Vie, and Armagnac at le Dôme in the Boulevard St Michel, until 1.00 am; and drifted back, catching (quite by chance) the very last Metro train, and so to bed. Next morning we read that there had been a huge demonstration going on all night in central Paris, which we had not even noticed.

So we separated, after much exchanging of addresses and hopes of returning. At £120 all-in for the week it represents very good value, as well as being a stimulating and uplifting experience; a chance to recharge the batteries and recapture, for a very short time, the invigorating carefree habits of student life, which we all once knew and which contrast so strikingly with the cares and responsibilities of day-to-day professional practice.

M. J. Kelly
Senior Surgical Registrar
Bristol Royal Infirmary
and
A Pollock
Consultant Haematologist
Birmingham General Hospital

Correspondence to: Mr M. J. Kelly, Consultant General Surgeon, Leicester General Hospital.

**Singapore Revisited**

I was glad of the opportunity to revisit Malaysia in August for the joint meeting of The Royal College of Physicians with the Singapore–Malaysia congress of medicine. When listening to papers, visiting hospitals or chatting informally over a meal, I was most impressed by the high standard of medicine and surgery being practised there. Many of the doctors had received some postgraduate training in the United Kingdom and hoped for more exchange visits and other links. I am sure that the standards of medicine in both countries would be improved by interchange of registrars and senior registrars, if this could be arranged.

Of particular interest to me was to see the changes in the country, having served in the Army there forty years ago. Very few of the old buildings remain – though the Raffles Hotel still serves an excellent gin sling! I could not recognize most of the roads as they have been changed into four or six lane highways. Needless to say, the Japanese and British camps where I lived after the war have been demolished, but the British Military Hospital Alexandra remains and is a great tribute to the British architect who designed it in the nineteen thirties. Most of China town has been demolished, the junks on the Singapore river have disappeared, being replaced by high-rise blocks of flats.

The indigenous population seemed as happy and friendly as before. By dint of their hard work they have achieved a high standard of living with very little unemployment. They are proud of their country, and seem constantly to want to improve it even more. Standards of hygiene are as high and one sees no litter as a large fine is imposed on anyone seen discarding rubbish of any kind (City of Bristol please take note!). One could not fail to be impressed by the development of Singapore as one of the top three ports of the world, largely because of the spirit of the people and the ethic of hard work.

Standards of health are high thanks to a safe and abundant water supply, good drains and an adequate supply of food based on a prosperous economy. The constant humid heat remains a problem, and it used to sap the energy of Europeans and locals alike. However, the widespread use of air conditioning in buildings and cars makes this much more tolerable. Now you need to put your coat on when going into an office or hotel as it so cool!

After a life-time spent in the practice of medicine one could not help posing the question: who has done most for the health of mankind? Is it the doctors, or is it the water and sewage engineers, builders, farmers and, more recently, the makers of central heating and air conditioning? I know where my vote would be cast, but in any case a country needs economic prosperity to pay for these services.

H. G. Mather

